# Current status of *Mycoplasma pneumoniae* infection in China

**DOI:** 10.1007/s12519-023-00783-x

**Published:** 2024-01-08

**Authors:** Chao Yan, Guan-Hua Xue, Han-Qing Zhao, Yan-Ling Feng, Jing-Hua Cui, Jing Yuan

**Affiliations:** Department of Bacteriology, Capital Institute of Pediatrics, No. 2 Yabao Road, Beijing, 100020 People’s Republic of China

*Mycoplasma pneumoniae* (*M. pneumoniae*) is one of the most important pathogens for community-acquired pneumonia worldwide, especially in children and adolescents [1, 2]. *M. pneumoniae* is easily transmitted through droplets or direct contact in densely populated, enclosed, or poorly ventilated environments. The incubation period is 1–3 weeks, and it is contagious from the incubation period to a few weeks after clinical symptom relief. *M. pneumoniae* infection can occur in any season, and there are differences in the epidemic seasons among regions in China [3]. The most suitable culture temperature for *M. pneumoniae* is between 35 ℃ and 37 ℃, and therefore, hot weather may make it survive longer in the environment and spread further. Infection is more common in autumn and winter in northern China, while it is more prevalent in summer and autumn in southern China. In addition to causing upper respiratory tract infections, *M. pneumoniae* can also cause bronchitis, pneumonia, as well as potentially fatal extra-pulmonary complications. Due to the strong mitigation measures, the number of children with community-acquired pneumonia caused by *M. pneumoniae* was significantly decreased after the coronavirus disease 2019 (COVID-19) pandemic [4]. However, *M. pneumoniae* was one of the most common pathogen in children infected with SARS-CoV-2 [5]. The incidence of Kawasaki disease was increased during the COVID-19 pandemic was accompanied by a high incidence of *M. pneumoniae* infection, especially in children less than 3 years old [6].

## Trends in *Mycoplasma pneumoniae* infection in children

Globally, *M. pneumoniae* infection occurs in a regional outbreak every 3–7 years, with each outbreak lasting for 1–2 years [7]. Large epidemics occurred in Asian and European countries between 2010 and 2012 [8–10]. There was a small peak from June to July 2023, and there was a slight decrease in August in China. With the arrival of the school season, the incidence of *M. pneumoniae* in September has shown an increasing trend, and there are reports of a high incidence of *M. pneumoniae* infection in various regions in China. According to data in Beijing, during this epidemic, the positive detection rate (by real-time PCR assay) of *M. pneumoniae* in outpatient patients can reach 25.4%, inpatients can reach 48.4%, and respiratory patients can reach as high as 61.1%. In the respiratory ward, more than 50% of hospitalized children are diagnosed with *Mycoplasma pneumoniae* pneumonia (MPP). With the increase in children with MPP, this epidemic has attracted widespread attention. The cause of this *M. pneumoniae* epidemic remains unclear.

In the past 30 years, P1 typing and multiple-locus variable-number tandem-repeat analysis (MLVA) are the most common genotyping methods for monitoring *M. pneumoniae*. P1 typing distinguished strains focused mainly on the sequence variations in the P1 gene (MPN140 to MPN142). *M. pneumoniae* can be divided into two main subtypes, type1 and type2, and many variants. P1 type 1 and 2 M*. pneumoniae* strains dominate alternately in cycles of ~ 10 years [11]. MLVA typing basing on the variations in the copy number of tandem repeats was developed in 2009 by Dégrange et al., and soon became a more powerful discriminatory method than P1 typing [12]. Five variable-number tandem-repeat loci (Mpn1 and Mpn13–16) were identified and revealed MLVA types from clinical strains. In recent years, some findings have suggested that in Japan and northern China, the genotypes of the predominant *M. pneumoniae* strains have shifted from P1 type 1 to type 2, or from type M4-5-7-2 to type M3-5-6-2. A periodic genotype shift in clinically prevalent *M. pneumoniae* between 2006 and 2019 was reported in Japan [13]. In 2011 and 2012, P1 type 1 was the predominant genotype, representing more than 80% of reported infections; however, type 2 strains increased in 2015 and 2016, and then dominated after 2017. Wang et al. reported that during 2016–2019, the proportion of type M4-5-7-2 strains decreased from 84.49 to 70.77%, while type M3-5-6-2 increased from 11.63% to 24.67% [14]. In our previous study, we reported that MLVA type M3-5-6-2 was correlated with severe MPP [15]. We have detected that the predominant genotype was P1 type 1 (85.7%) and MLVA type M4-57-2 (67.1%) since July in Beijing. However, the relationship between genotype and the occurrences of current *M. pneumoniae* outbreak in China is unknown. We hypothesize that it is related to genotype instability of the currently popular *M. pneumoniae* strains.

## Increased macrolide resistance rate of *Mycoplasma pneumoniae*

The recently published 2023 edition of the National Health Commission’s “Guidelines for the Diagnosis and Treatment of *Mycoplasma pneumoniae* in Children” recommends doxycycline as alternative drugs for the treatment of MMP in children. Macrolides are still used in China as the first-line antibiotics for the treatment of MPP in Children [16]. Macrolides restrained bacterial growth by binding of the 23S rRNA to inhibit protein synthesis. However, macrolide-resistant *Mycoplasma pneumoniae* (MRMP) have emerged widely in Asian countries since 2000 and are increasing rapidly, which representing approximately 80%–90% of MPP cases in China and Japan [17–19]. A correlation between the specific genotype P1 type2, MLVA type M4-5-7-2 and macrolide resistance has been reported [20–22]. Notably, with the shift in the dominant genotype of *M. pneumoniae*, the macrolide resistance rate in type M3-5-6-2 strains has drastically increased, from 60% to 93.48% [14]. However, the incidence of MRMP has decreased in Japan with the genotype shift [13]; the detection rate of MRMP is very low in Europe and the United States [23, 24]. Therefore, the frequency of macrolide usage is correlated with differences in drug resistance in different countries [25]. These results reveal that the current increased macrolide resistance rate of *M. pneumoniae* may be simultaneously correlated with genotype shifting and macrolide usage.

Multiple hospitals have observed that the current outbreak of *M. pneumoniae* infection is predominantly caused by MRMP. The mutation rate of macrolide-resistant genes in 23S rRNA is up to 97.1% in Beijing, significantly higher than previous reported data (around 90%). MRMP often manifests as high fever, severe cough, and poor mental health, resulting in a long course of disease, a long hospital stay, and poor prognosis.

## Clinical characteristics of *Mycoplasma pneumoniae* pneumonia in the current outbreak

The clinical characteristics are characterized by younger age, increased hypoxemia, local lung damage, worsening systemic inflammation, and increased co-infection. During the current outbreak in China, MPP has commonly been seen in children aged 5 and above. However, compared to previous years, this outbreak has shown a younger age trend in children under 3 years. The principal manifestations of *M. pneumoniae* infection are fever and cough, with moderate-to-high fever being common, but may present as low-grade fever or no fever at all. Some children may experience fever accompanied by symptoms, such as chills, headaches, chest pain, and chest tightness; others may experience wheezing, and in severe cases, shortness of breath and difficulty breathing may occur. The characteristic *M. pneumoniae* cough is relatively severe, often presenting with paroxysmal dry cough in the early stages, and may be accompanied by phlegm in later stages. The color of the phlegm is generally white and sticky, with some cases involving yellow phlegm, and occasionally with blood in the phlegm. The cough shows a gradually worsening trend, and some children may develop pertussis-like symptoms, with a disease course lasting 2 weeks or longer.

The course of MPP develops rapidly, and it can progress to pneumonia after 2–3 days of high fever. Chest X-rays or computer tomography scans often show lobar pneumonia, with some patients presenting with “white lung” abnormalities. MPP is often associated with pleural effusion and atelectasis, and it may also lead to pneumothorax or necrotizing pneumonia. MPP has a wide range, a long course, and a severe condition. These infections are commonly complicated by bacterial (*Streptococcus pneumoniae*) or viral (*adenoviruses*) infections, making the condition worse.

## Treatment for macrolide-resistant *Mycoplasma pneumoniae*

The key of MPP treatment is early identification and treatment of severe MPP and fulminant MPP, and the optimal treatment window is within 5–10 days after fever. Individualized treatment plans should be developed based on diagnosis. Severe patients should adopt comprehensive treatment with different focuses (combination of anti-infection, glucocorticoids, bronchoscopy, anticoagulation, etc.), focusing not only on mixed infections, but also accurately identifying and treating excessive inflammatory reactions and cytokine storms [26]. Typically, penicillin and cephalosporins are ineffective in the treatment of MPP, while macrolide antibiotics, including azithromycin, clarithromycin, erythromycin, roxithromycin, and acetylkitasamycin, are commonly used in children with MPP. However, due to the increasing proportion of MRMP among current infections, the treatment effect of erythromycin and azithromycin on MPP is not satisfactory. For children under 8 years old with lobar pneumonia, to accelerate the absorption of pneumonia and reduce pneumonia complications and sequelae, tetracycline antibiotics including doxycycline and minocycline may be used. For children who do not respond well to conventional treatment or who have been diagnosed with macrolide-unresponsive MPP, refractory MMP, or severe MPP, the use of quinolone antibiotics, including levofloxacin and moxifloxacin, may be considered. For acute-onset, rapidly developing and severe MPP, especially severe or refractory pneumonia, the use of systemic glucocorticoids can be considered. For critically ill children suspected of having a mucus blockage or plastic bronchitis, bronchoscopy and alveolar lavage should be performed as soon as possible. If there is a tendency toward high coagulation parameters, heparin anticoagulant therapy should be used as soon as possible. When MPP is accompanied by bacterial or viral infections, medication should be used in combination. What is important is that when using medication beyond the instructions (tetracycline and quinolone antibiotics), it is necessary to fully evaluate the pros and cons and obtain informed consent from parents.

## Improving the accuracy of early diagnosis and preventing the occurrence of severe pneumonia

The following indicators indicate a risk of *M. pneumoniae* infection developing into severe or critical illness: persistent high fever within 72 hours after treatment; symptoms of infection and poisoning; imaging evidence of disease progressing rapidly, with infiltration into multiple lung lobes; significant increases in inflammatory indicators, with earlier appearance portending a more severe condition; difficulty in alleviating or progressing hypoxemia and dyspnea after treatment; existence of underlying diseases, including asthma and primary immunodeficiency disease; and delayed treatment with macrolide antibiotics [26].

To prevent the occurrence of severe pneumonia, it is important to improve the accuracy of early diagnosis of MPP, especially MRMP infection. *M. pneumoniae* culture is the gold standard for diagnosis, but not a good choice for rapid clinical diagnosis owing to the special culture conditions and slow growth. *M. pneumoniae* nucleic acid detection, including MP-DNA or MP-RNA detection, with high sensitivity and specificity, is suitable for early diagnosis of MPP. New diagnostic methods such as loop-mediated isothermal amplification, recombinase-aided amplification, and droplet digital PCR can be chose to for detection of *M. pneumoniae* in clinical specimens. The *M. pneumoniae* antibody immunoglobulin M (IgM) generally appears 4–5 days after infection and may be used as a diagnostic indicator for early infection, but antibody results must be combined with clinical and imaging features for comprehensive analysis.

In summary, since June 2023, several regions in China have experienced an early peak of *Mycoplasma pneumoniae infection in children*. In September, there was a significant increase in *M. pneumoniae* infection cases, with severe clinical manifestations. The strains are mainly macrolide-resistant *M. pneumoniae*. MRMP often leads to more severe clinical symptoms, increasing the difficulty of treatment. Therefore, timely diagnosis and reasonable antibiotic application are crucial for the current outbreak.

## Data Availability

Not applicable.

## References

[1] Parrott GL, Kinjo T, Fujita J (2016). A compendium for *Mycoplasma pneumoniae*. Front Microbiol.

[2] Waites KB, Xiao L, Liu Y, Balish MF, Atkinson TP (2017). *Mycoplasma pneumoniae* from the respiratory tract and beyond. Clin Microbiol Rev.

[3] Wang X, Li M, Luo M, Luo Q, Kang L, Xie H (2022). *Mycoplasma pneumoniae* triggers pneumonia epidemic in autumn and winter in Beijing: a multicentre, population-based epidemiological study between 2015 and 2020. Emerg Microbes Infect.

[4] Zhang LN, Cao L, Meng LH (2022). Pathogenic changes of community-acquired pneumonia in a children's hospital in Beijing, China before and after COVID-19 onset: a retrospective study. World J Pediatr.

[5] Li W, Wang BH, Chen BH, Sun Y, Li L, Xiang WQ, et al. Coinfection of SARS-CoV-2 Omicron variant and other respiratory pathogens in children. World J Pediatr. 2023. 10.1007/s12519-023-00744-4. 10.1007/s12519-023-00744-4 37540450

[6] Ding YY, Ren Y, Qin J, Qian GH, Tang YJ (2021). Clinical characteristics of Kawasaki disease and concurrent pathogens during isolation in COVID-19 pandemic. World J Pediatr.

[7] Yamazaki T, Kenri T (2016). Epidemiology of *Mycoplasma pneumoniae* infections in Japan and therapeutic strategies for macrolide-resistant *M. pneumoniae*. Front Microbiol.

[8] Chalker V, Stocki T, Mentasti M, Fleming D, Harrison T (2011). Increased incidence of *Mycoplasma pneumoniae* infection in England and Wales in 2010: multiocus variable number tandem repeat analysis typing and macrolide susceptibility. Euro Surveill.

[9] Nir-Paz R, Abutbul A, Moses AE, Block C, Hidalgo-Grass C (2012). Ongoing epidemic of *Mycoplasma pneumoniae* infection in Jerusalem, Israel, 2010 to 2012. Euro Surveill.

[10] Sun H, Xue G, Yan C, Li S, Zhao H, Feng Y (2017). Changes in molecular characteristics of * Mycoplasma pneumoniae * in clinical specimens from children in Beijing between 2003 and 2015. PLoS ONE.

[11] Kenri T, Okazaki N, Yamazaki T, Narita M, Izumikawa K, Matsuoka M (2008). Genotyping analysis of *Mycoplasma pneumonia*e clinical strains in Japan between 1995 and 2005: type shift phenomenon of *M. pneumoniae* clinical strains. J Med Microbiol.

[12] Dégrange S, Cazanave C, Charron A, Renaudin H, Bébéar C, Bébéar CM (2009). Development of multiple-locus variable-number tandem-repeat analysis for molecular typing of *Mycoplasma pneumoniae*. J Clin Microbiol.

[13] Kenri T, Suzuki M, Sekizuka T, Ohya H, Oda Y, Yamazaki T (2020). Periodic genotype shifts in clinically prevalent *Mycoplasma pneumoniae* strains in Japan. Front Cell Infect Microbiol.

[14] Wang Y, Xu B, Wu X, Yin Q, Wang Y, Li J (2021). Increased macrolide resistance rate of M3562 *Mycoplasma pneumoniae* correlated with macrolide usage and genotype shifting. Front Cell Infect Microbiol.

[15] Yan C, Xue G, Zhao H, Feng Y, Li S, Cui J (2019). Molecular and clinical characteristics of severe *Mycoplasma pneumoniae* pneumonia in children. Pediatr Pulmonol.

[16] Bradley JS, Byington CL, Shah SS, Alverson B, Carter ER, Harrison C, Pediatric Infectious Diseases Society and the Infectious Diseases Society of America (2011). The management of community-acquired pneumonia in infants and children older than 3 months of age: clinical practice guidelines by the Pediatric Infectious Diseases Society and the Infectious Diseases Society of America. Clin Infect Dis.

[17] Meyer Sauteur PM, Unger WW, Nadal D, Berger C, Vink C, van Rossum AM (2016). Infection with and Carriage of *Mycoplasma pneumoniae* in Children. Front Microbiol.

[18] Pereyre S, Goret J, Bébéar C (2016). *Mycoplasma pneumoniae*: current knowledge on macrolide resistance and treatment. Front Microbiol.

[19] Zhao F, Lv M, Tao X, Huang H, Zhang B, Zhang Z (2012). Antibiotic sensitivity of 40 *Mycoplasma pneumoniae* isolates and molecular analysis of macrolide-resistant isolates from Beijing. China Antimicrob Agents Chemother.

[20] Qu J, Yu X, Liu Y, Yin Y, Gu L, Cao B (2013). Specific multilocus variable-number tandem-repeat analysis genotypes of *Mycoplasma pneumoniae* are associated with diseases severity and macrolide susceptibility. PLoS ONE.

[21] Zhao F, Liu G, Wu J, Cao B, Tao X, He L (2013). Surveillance of macrolide-resistant *Mycoplasma pneumoniae* in Beijing, China, from 2008 to 2012. Antimicrob Agents Chemother.

[22] Yan C, Sun H, Lee S, Selvarangan R, Qin X, Tang YW (2015). Comparison of molecular characteristics of *Mycoplasma pneumoniae* specimens collected from the United States and China. J Clin Microbiol.

[23] Kogoj R, Mrvic T, Praprotnik M, Kese D. Prevalence, genotyping and macrolide resistance of *Mycoplasma pneumoniae* among isolates of patients with respiratory tract infections, Central Slovenia, 2006 to 2014. Euro Surveill. 2015;20(37). 10.2807/1560-7917.ES.2015.20.37.30018. 10.2807/1560-7917.ES.2015.20.37.30018 26536357

[24] Xiao L, Ratliff AE, Crabb DM, Mixon E, Qin X, Selvarangan R (2020). Molecular Characterization of *Mycoplasma pneumoniae* Isolates in the United States from 2012 to 2018. J Clin Microbiol.

[25] Morozumi M, Iwata S, Hasegawa K, Chiba N, Takayanagi R, Matsubara K, Acute Respiratory Diseases Study Group (2008). Increased macrolide resistance of *Mycoplasma pneumoniae* in pediatric patients with community-acquired pneumonia. Antimicrob Agents Chemother.

[26] National Health Commission of the People's Republic of China. Guidelines for the diagnosis and treatment of *Mycoplasma pneumoniae* pneumonia in Children (2023 edition). Inter J Epidemiol Infect Dis. 2023;50:79–85.

